# A Case Report of Uterine Torsion: An Obstetric Emergency During Pregnancy

**DOI:** 10.7759/cureus.52538

**Published:** 2024-01-18

**Authors:** Vidya Gaikwad, Sneha Aramandla, Suhas Gaikwad

**Affiliations:** 1 Obstetrics and Gynecology, Dr. D. Y. Patil Medical College, Hospital and Research Centre, Dr. D. Y. Patil Vidyapeeth (Deemed to be University), Pune, IND

**Keywords:** oligohydramnios, insufficiency, fetoplacental, uteroplacental, uterine torsion

## Abstract

Uterine torsion is an exceedingly rare obstetric emergency representing pelvic organ torsion, characterized by the uterus rotating more than 45 degrees around the longitudinal axis. This torsion predominantly occurs at the junction of the cervix and uterine corpus. Albeit the infrequent prevalence, this condition can arise in any reproductive group. Oligohydramnios is defined as an amniotic fluid volume of 2 cm or less in the single deepest vertical pocket. During pregnancy, uterine torsion is known to be associated with severe maternal and perinatal consequences encompassing placental abruption, maternal mortality, and intrauterine fetal demise. Here, we present a specific case of a woman who experienced uterine torsion during pregnancy, leading to complications such as uteroplacental and fetoplacental insufficiency, severe fetal growth restriction, and persistent oligohydramnios throughout the pregnancy.

## Introduction

Uterine torsion, first documented in 1861, remains an exceptionally rare obstetric condition, with just over 200 cases reported in the last century [1]. This condition is characterized by the uterus undergoing a rotation of more than 45 degrees and up to 180 degrees around its longitudinal axis [2]. Notably rare, many obstetricians and gynecologists may only encounter this obstetrical emergency once in their professional careers [3]. The primary indicators of uterine torsion during pregnancy typically include abdominal pain, changes in fetal heart rate, and a failure of cervical dilatation [4]. Detecting uterine torsion is more challenging in asymptomatic cases [5]. Abdominal pain in pregnancy may not readily raise suspicion of uterine torsion, yet severe acute instances can lead to complications such as placental abruption, maternal mortality, and intrauterine fetal demise [6,7]. Given its nonspecific and elusive symptoms that may mimic other obstetric complications, particularly placental abruption, diagnosing uterine torsion is intricate and often deferred until the patient undergoes operative intervention [8].

## Case presentation

A 20-year-old, primigravida, at 32 weeks and four days gestational age, presented to the labor room with complaints of lower abdominal pain for two days. While her symptoms were initially mild, her pain escalated significantly three to four hours prior to presentation. Notably, she was experiencing occasional elevated blood pressure during the current pregnancy, for one month, for which she was started on oral labetalol 100 mg twice daily for the past two days. She had a history of hematinic injections at 16 weeks of gestation for the treatment of anemia with hemoglobin of 8g/dL and another hospitalization for threatened preterm labor, at 24 weeks of gestation. Subsequently, she had another admission at 28 weeks of gestation for the management of mild oligohydramnios.

Upon examination, her blood pressure measured 130/90 mmHg and her pulse rate was 90 beats per minute. She was afebrile, with a temperature of 98.2F, oxygen saturation of 99% on room air, and a respiratory rate of 16 breaths per minute. On palpation, the height of the uterus corresponded to 26 weeks of gestation with a lag of six weeks. The uterus was well relaxed with no tenderness, and the fetal heart rate was 140 beats per minute. A per vaginal examination was done, which revealed dark, altered blood, while the cervix was not dilated and uneffaced. Upon admission, the necessary blood investigations including pregnancy-induced hypertension (PIH) profile were done, which turned out to be normal, with a hemoglobin of 11.5g/dL. An ultrasonography was performed, which reported a single, live, intrauterine gestation of 25 weeks, indicative of stage 4 fetal growth restriction, an AFI of 0 cm suggestive of anhydramnios, placenta located at the fundus, fetal heart rate of 154 beats per minute, EFW of 636 grams (<1 percentile), a biophysical profile of 4/10, and Doppler findings indicative of uteroplacental and fetoplacental insufficiency. Therefore, she was taken up for emergency Lower segment cesarean section after counseling.

**Figure 1:**
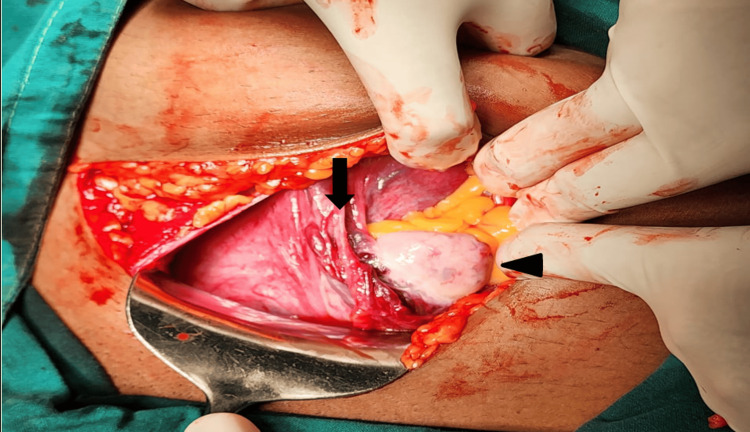
Intraoperative picture showing uterine torsion Intraoperative image showing the uterus (arrow) and left-sided ovary (arrowhead) visible at the center.

Intraoperative findings showed the uterus to have been dextrorotated to over 90 degrees, with the left ovary and round ligament was visible beyond the center of the abdomen (Figure 1). The uterine artery was found to be tortuous. 

**Figure 2:**
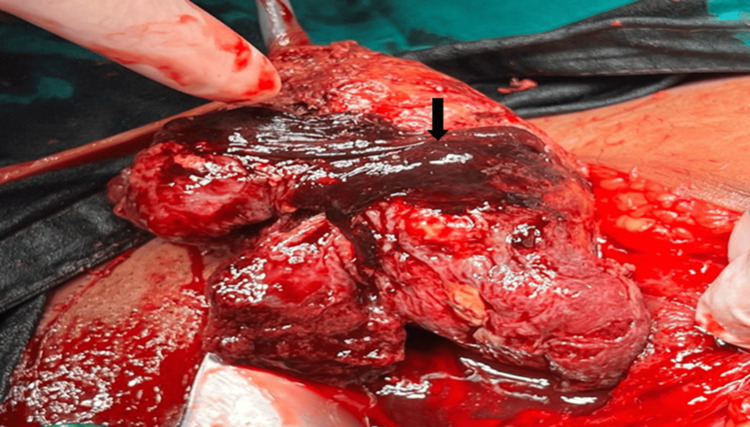
Intraoperative image showing concealed abruption Concealed abruption of the placenta in the uterus (arrow) was noted during the procedure after delivery of the placenta that was located at the fundus.

Notably, there were no observed uterine anomalies, fibroids, or ovarian pathologies. The uterus did not respond to gentle palpation for rotation. However, the senior obstetrician successfully manually rotated the uterus through manipulation, enabling an incision to be made toward the left from the midline. There was no amniotic fluid, and the asphyxiated baby was seen in a transverse lie and delivered through breech extraction. The baby was covered with thick meconium and had a poor APGAR score of 3/10 at one minute after birth, and a score of 5/10 at five minutes after birth, with a birth weight of 700 grams. Neonatal resuscitation was done, and the baby was shifted to NICU. Active management of the third stage of labor was practiced, by giving injection oxytocin 10 IU intramuscularly, after delivery of the baby, to remove the placenta that was in the upper segment. A concealed abruption was also noted (Figure 2).

The postoperative period was uneventful with no puerperal complications. Hemoglobin was 11 g/dL postoperatively. The patient recovered well and was discharged on post-operative day seven after suture removal was done. Unfortunately, the baby died on day eight due to cardiorespiratory arrest and an extremely low birth weight.

## Discussion

Pregnancy elevates the congenital and physiologic rotations and obliquities of the uterus, contributing to the occurrence of uterine torsion in gravid uteri compared to nongravid ones [9]. Observable asymmetry resulting from congenital or acquired deformities such as uterine fibroids, uterine anomalies, or pelvic adhesions may predispose to uterine torsion, most commonly during the third trimester. Additionally, structural weaknesses (whether developmental or acquired) and angulation in the isthmic region can also be factors leading to torsion [10]. Notably, the majority of literature concerning the condition comes from individual case reports; hence, the precise etiology of uterine torsion remains unknown and appears unrelated to maternal age, parity, and gestational age. Intriguingly, Jensen’s series identified the transverse lie as the most frequent abnormal fetal presentation (72%) [11]. However, recent reviews have suggested that uterine torsion typically occurs within a normal pelvis and is not associated with any pelvic pathology. Nevertheless, this particular case comes under the 30% of cases that occur where a discernable cause is not identified [12]. In this condition, the mother and the fetus are exposed to substantial risk. Maternal hemodynamic shock and complete or partial placental abruption are the most commonly associated maternal complications [9,10]. On the other hand, fetal complications associated include fetal hypoxia, fetal antepartum hemorrhage, and the risk of fetal demise. Imaging modalities such as USG, CT, and MRI can offer valuable diagnostic insights to a certain extent [10]. Unsuccessful attempts at detorsioning the uterus may lead surgeons to deliver through a posterior low transverse hysterotomy [13].

In this case, a primigravida presented with persistent lower abdomen pain, severe fetal growth restriction, and anhydramnios. The diagnosis was made during surgery, revealing a surprising intraoperative finding such as uterine torsion with concealed abruption placenta. The potential link between torsion-induced uteroplacental insufficiency and subsequent severe fetal growth restriction cannot be ruled out because of the limited existing literature on the subject.

## Conclusions

In pregnant women, uterine torsion is an infrequent event, often eluding diagnosis, and if overlooked, can result in severe consequences. Therefore, when a woman, regardless of age, presents with acute abdominal pain and vaginal bleeding, healthcare professionals should include uterine torsion in their list of potential differential diagnoses. The case outlined in this report exemplifies how uterine torsion can lead to significant complications during pregnancy, posing a substantial risk of morbidity and mortality for both the mother and the baby. Once diagnosed, the primary goal of management is to restore normal uterine anatomy and address any complications arising from uterine torsion.
